# Predictors of the return to work for pregnant employees on preventive leave: Patients from an occupational medicine consultation in Switzerland

**DOI:** 10.1371/journal.pone.0300686

**Published:** 2024-03-22

**Authors:** Karine Moschetti, Loïc Brunner, Alessia Abderhalden-Zellweger, Isabelle Probst, Saira-Christine Renteria, Julien Vonlanthen, Peggy Krief

**Affiliations:** 1 Center for Primary Care and Public Health (Unisanté), Department of Epidemiology and Health Systems (DESS), University of Lausanne (UNIL), Lausanne, Switzerland; 2 Center for Primary Care and Public Health (Unisanté), Department of Health, Work and Environment (DSTE), University of Lausanne (UNIL), Lausanne, Switzerland; 3 School of Health Sciences (HESAV), University of Applied Sciences and Arts Western Switzerland (HES-SO), Lausanne, Switzerland; 4 Centre Hospitalier Universitaire Vaudois, (CHUV), University of Lausanne (UNIL), Lausanne, Switzerland; Shahrood University of Medical Sciences, ISLAMIC REPUBLIC OF IRAN

## Abstract

According to the Swiss legislation on maternity protection in the workplace (OProMa), if pregnant workers are exposed to occupational hazards and no protective measures are taken, a gynecologist will prescribe a certificate of preventive leave and the women must stop working. Returning to work is only possible if job adjustments are made. This study aims to evaluate the burden of absences on companies and to examine the predictors of the return to work for pregnant workers on preventive leave, by examining both the probability of return to work and the time required to return to work. The study sample includes data on 258 workplaces of pregnant workers on preventive leave, collected during an occupational medicine consultation aimed at supporting the implementation of the OProMa. Information is available on the worker (age, date of consultation), the hazards to which she is exposed, the company’s knowledge of the OProMa and whether a risk analysis exists. Descriptive statistics and multivariate regression analysis are carried out. In 58% of the workplaces, it was not possible to return to work before the end of the pregnancy. This corresponds to an average absence of 4.5 months. In 42% of the workplaces, a return to work was possible thanks to workplace adaptations. A conforming risk analysis and a full knowledge of the OProMa for companies, and an early visit to the occupational medicine consultation for workers are good predictors of the likelihood of returning to work. Younger age and exposure to certain types of risks are factors that influence the duration of preventive leave. The implementation of OProMa in Switzerland poses serious challenges, but early identification of occupational hazards and practices that anticipate compliance with the law in the company increase the return to work in safety for pregnant workers.

## Introduction

In the countries of the Organization for Economic Cooperation and Development (OECD), women make up an increasingly large share of the labor force. In 2019, almost 71% of mothers were in the labor force [1]. Due to physical changes caused by pregnancy and because the intrauterine development is a sensitive period, pregnant women may have special needs, especially those who continue to work during their pregnancy.

There is a large body of literature on the adverse health effects of occupational hazards (chemicals, biological, prolonged standing, night work, etc.) on pregnant workers and their future children [2, 3]. Based on this literature, which reports on reprotoxic risks of certain working conditions, legislators in many countries have enacted a number of legal measures to protect pregnant workers.

At the European level, the Article 5 of the current European Union Directive 92/85/ECC aims at “implementing measures to encourage improvements in the safety and health at work of pregnant workers…” [4]. In most European countries there are laws on maternity at work which specify the working conditions that may pose an increased risk to pregnant women and dictate how these risks should be assessed and managed [5–7]. At the Swiss level, the legal protection of pregnant workers, derived from the Federal Labor Law, is called Ordinance on Maternity Protection (OProMa) [8]. It came into force in 2001 and was revised in 2008 and 2015. In particular, it details the respective duties and rights of employers, gynaecologist, pregnant workers and occupational health specialists [8].

In practice, the application of these legislations does not appear to be homogenous, and workplaces are not systematically adapted to the needs of pregnant workers and to the protection of their health. In the context of French-speaking Switzerland, a recent study carried out in two occupational sectors shows that only 12% of workers in the health sector and 2% in the food industry benefit from the protection in accordance with the Swiss legislation [9]. Companies apply the legislation in a heterogeneous/partial manner and gynecologists dot not always feel competent in occupational health issues to assess the hazardousness of working conditions of pregnant women [5, 9–11]. At the international level, publications underline the heterogeneous applications of the legislations surrounding maternity protection at work. On the one hand, one observes substantial volumes and variability of absences from work during pregnancy. For instance, in Denmark, Norway, France, and Spain, studies reported that pregnant workers have higher levels of absence than non-pregnant workers [12–14]. On the other hand, not all these absences are caused by pathologic problems or symptoms related to pregnancy but by inadequate working conditions [15]. Several studies show a strong association between strenuous working conditions (such as work postures, carrying heavy load, shift work, night work, increasing working hours, and occupational strain) and work interruptions [13, 16–21]. Finally, publications demonstrate that with work adaptations, absences of pregnant women can be reduced. Although the literature on job accommodation during pregnancy and its effects on absences from work and absence duration is limited, few studies provide insight into the expected effects on absences. Based on a survey of several samples of pregnant women in Scandinavian countries, a couple of studies highlighted the strong association between the need of job accommodation and absence from work. Using a Norwegian representative survey focusing on working conditions during pregnancy, results showed that women who reported a need for accommodation but did not receive it had a higher rate of absence from work compared with those who received it [22]. Still in Norwegian context, receiving accommodations was associated with a lower risk of absence [23]. Such findings were confirmed in a context of absence from work due to low back pain among Danish women [24]. Although the job accommodations did not completely prevent work interruptions, longitudinal analyses show that the duration of absence was 1 or 2 weeks shorter for pregnant workers who received accommodations than for those who did not [12]. Finally, job adjustments were also associated with an increased likelihood of returning to work among those who started being absent [23].

Absence from work raises number of challenges for employers and employees as well for society as a whole. Absences from work are costly. For employers, an employee’s absence often requires a replacement and sometimes means a disruption in the organization of work, which can reduce productivity, increase the workload of other employees in the company, and negatively affect the performance or quality of services provided. The literature on sickness absence is well documented [25, 26]. In the case of maternity, studies tend to focus on the post-natal period rather than the pre-natal period [27, 28]. Few studies deal with absence from work during pregnancy. Two types of absences can be distinguished: sick leave (due to the employee’s health condition or pregnancy pathology) and preventive leave (aimed at protecting the employee from dangerous or strenuous exposures). However, the appropriate use of one or the other category is not easy. Recent research in French-speaking Switzerland [29] has shown that when gynecologists perceive their patients to be exposed to occupational hazards, they are more likely to place them on sick leave than to prescribe preventive leave to limit these risks. It is not uncommon for sick leave to be used instead of preventive leave, even though most legislations distinguish both cases [18, 21]. Unfortunately, prescribing sick leave may simply make potential exposures to occupational hazards or strenuous activities invisible. While work interruptions–sick leaves—can be used to prevent hazardous exposures for pregnant workers when necessary, they can also reduce income and affect mental health and future social relationships at work [30]. Failure to enforce the law may lead to adverse maternal and neonatal issues if the woman continues to work despite the hardship or danger, or to work interruptions.

Given this, there is scope to reduce some absenteeism by adjusting working conditions during pregnancy. It is therefore important to increase knowledge about the relationship between absenteeism and workplace accommodation during pregnancy. With this objective in mind, the present study aims 1) to assess the burden on companies of absenteeism among pregnant workers who have received a certificate of preventive leave from their gynaecologist, due to exposure to strenuous and hazardous occupational activities, and 2) to investigate the determinants of their return to work in safe conditions. To this end, two outcomes will be analyzed: the probability of returning to work and the time required to do so. Pregnant workers, and work environment characteristics and employer factors are tested as predictors of return to work during pregnancy.

## Method

### Setting

In Switzerland, the OProMa regulates the obligations of employers, gynecologist, and the rights of pregnant workers [8]. It specifies which working conditions pose a risk to pregnant workers and future mothers and how these risks should be managed. In compliance with the law, the employer must call in an occupational health specialist to carry out a risk analysis. The occupational health specialist documents both the occupational risks and the recommended protective measures in a written risk analysis report. The employer must then adapt the workplace in accordance with the instructions given in the risk analysis, and if this is not possible, provide an alternative job position that does not involve hazardous or strenuous work during the pregnancy. The costs of conducting the risk analysis and implementing the adjustments are borne by the company. In addition, during pregnancy consultations, the employee’s gynecologist determines whether her patient performs hazardous work activities and also reviews the risk analysis report. If the pregnant worker is exposed to occupational hazards that are not treated with specific preventive measures, the gynecologist issues a preventive leave certificate. This document prohibits the employer from keeping the employee on the job until the risk analysis and workplace adaptations have been carried out. During this period, the employer must pay the employee compensation equal to 80% of her income. Compensation continues through the end of the preventive leave, which means it is paid until necessary accommodations are made to ensure the employee can safely return to work. The length of the preventive leave and the subsequent ability to return to work will depend on the specific work situation and the employer’s ability to provide a safe environment for the employee.

In the canton of Vaud, in the French speaking part of Switzerland, a specialized occupational medicine consultation—namely PregOH-consultation—was introduced in 2015. Gynecologists can refer their patients to this innovative consultation if they suspect an occupational hazard and the company does not have an in-house occupational health physician. The PregOH-consultation, carried out by an occupational health physician, provides advice to both employees and employers on maternity protection in the workplace. In practice, the consultation informs workers about their legal rights, identifies risky exposures to which they may be exposed, informs employers about their legal obligations and helps them navigate through the process of risk analysis and workplace adjustments (see details in [29]).

### Data and variables

This study uses data collected from pregnant workers who visited the PregOH-consultation between 2015 and 2021. Information on the characteristics of pregnant workers, their work environment, their work-pregnancy reconciliation pathways (e.g., date of visit to the PregOH-consultation, start date of preventive leave, end date of preventive leave), and of their employers were collected.

The outcome of interest–the dependent variable—is the return to work of pregnant workers on preventive leave. To investigate this, we construct two outcome variables: a binary variable indicating whether or not the return to work occurred or not, and a count variable indicating the number of days required for the pregnant workers to return to work, when it occurred. This distinction allows us to model separately the probability of returning to work and the time (expressed in days) required to do so, if possible. The number of days corresponds to the duration of preventive leave and mainly reflects the time needed to make job adjustments.

As explanatory variables, we use the age of the workers, the time when the pregnant workers were referred to the PregOH-consultation (within the first 3 months of pregnancy or later), and the presence and nature of hazard exposures to which the pregnant workers were exposed. The exposures are identified and classified according to the Swiss labor law and the OProMa and, grouped into 9 categories [31]. For each of these categories, a dummy variable is created to indicate whether the exposure was present or not. Regarding the companies, two variables are considered: their knowledge of the OProMa and whether, or not a risk analysis was performed. Knowledge of the OProMa was assessed by the occupational health physician who performed the consultation using a three-modalities-score based on the employer’s answers to several questions about the law (see S1 Appendix for details on the criteria used to generate the score). From this score, we generate dummy variables distinguishing between the following three modalities: having “total knowledge”, “partial knowledge”, or “no knowledge” of the legislation. In addition, the occupational health physician of the PregOH-consultation confirmed or not that the employer/company has a risk analysis that complies with the law. In this respect three modalities were identified: “having a conforming risk analysis report”, “having a non-conforming risk analysis report”, or “not having carried out any risk analysis”. A non-conforming risk analysis report means that it is unsatisfactory, provided the work situation; the result of the risk analysis or the recommended protective measures may be incomplete, or the risk analysis has not been carried out by the qualified professional. In the analysis, we account for these three situations through dummy variables.

Information on 328 workstations from pregnant workers who were referred to the PregOH-consultation were collected. After removing missing values for the variables, we consider in the analysis, our study sample includes data on 258 work situations corresponding to 251 pregnant workers on preventive leave and 234 related employers.

### Statistical analysis

The unit of the analysis is the workstation. Descriptive statistics are presented for the dependent and independent variables to examine the characteristics of the sample. Return to work is then modelled using two regression models. First, the probability of returning to work is modelled using logistic regression. Second, the number of days required to return to work is modelled for those who returned to work using a zero-truncated negative binomial regression. The models allow for differences in the effect of specific variables on the probability of returning to work versus the number of days required to return to work. Analyses are conducted using R software version 4.0.2.

### Ethical considerations

The Human Research Ethics Committee of the Canton Vaud (CER-VD) has certified that the research study protocol associated with this study falls outside of the field of application of the Swiss Federal Act on Research Involving Humans (Req-2020-01320). The participation in the study was voluntary. "All participating pregnant workers were informed about the research objectives, the standards of confidentiality regarding the use of the data, and they signed a consent form at the end of the PregOH consultation. If the pregnant worker did not consent to her employer being contacted, the occupational physician refrained from contacting the employer, resulting in no information being collected. Employers gave verbal consent to participate in the survey and to record the telephone call. The data were analyzed anonymously.”

## Results

### Descriptive statistics

Tables 1 and 2 describe the characteristics of the study sample—explanatory and dependent variables. In the 258 workstations studied, 65% were occupied by women between 26 and 34 years of age and 18% by women over 35 years of age. In 21.3% of the workstations, the workers were referred to the PregOH-consultation by their gynecologist during the first 3 months of their pregnancy.

**Table 1:** Summary statistics of the explanatory variables.

Explanatory variables	% (N)
**Pregnant workers characteristics**	
Aged 19–25	16.67 (43)
Aged 26–34^a^	65.50 (169)
Aged 35–46	17.83 (46)
visited the PregOH-consultation during the first 3 months	21.32 (55)
**Nature of risk exposure of workstations**	
Postures	95.35 (246)
Handling heavy load	87.98 (227)
Chemicals	62.40 (161)
Microorganism	36.43 (94)
Physical	31.01 (80)
Noise	13.57 (35)
Vibration	9.69 (25)
Working pace	8.14 (21)
Ionizing Radiation and Electromagnetic field	4.26 (11)
**Employer characteristics**	
Workstations for which company has total knowledge of the OProMa	6.98 (18)
Workstations for which company has partial knowledge of the OProMa	52.71 (136)
Workstations for which company has no knowledge of the OProMa^a^	40.31 (104)
Workstations for which company has a conforming risk analysis report	3.88 (10)
Workstations for which company has a non-conforming risk analysis report	9.30 (24)
Workstations for which company has not performed any risk analysis^a^	86.82 (224)

^a^ Reference category in regression models

**Table 2:** Summary statistics on the proportion of workstations for which pregnant workers returned to work and time necessary to do so.

**Proportion of workstations for which the return to work was possible**						**% (N)**	
• among the whole sample (N = 258)						41.47 (107)	
• when there is total knowledge of the OProMa (N = 18)						72.22 (13)	
• when there is partial knowledge of the OProMa (N = 136)						44.11 (60)	
• when there is no knowledge of the OProMa (N = 104)						32.69 (34)	
• when there is a conforming risk analysis report (N = 10)						90 (9)	
• when there is a non-conforming risk analysis report (N = 24)						62.5 (15)	
• when there is no realized any risk analysis (N = 224)						37.05 (83)	
**Time in days necessary to return to work (N = 107)**							
**Mean**	**Standard Deviation (SD)**	**Min**	**25th percentile**	**Median**	**75th percentile**		**Max**
26.72	28.83	1	9.50	18	34.50		168

Exposures were variable and depended on the type of work. In the sample, all pregnant women were exposed to at least one type of exposure and on average they were exposed to 3.5 types of exposure. About 95.3% of the jobs involved strenuous postures, 87.9% of the jobs involved load handling, while noise, vibration and working pace, were present respectively in 13.6%, 9.7%, and 8.1%, of the jobs, respectively.

For more than half of the workstations (59.7%), the employers declared having either partial or total knowledge of the OProMa. For 3.8% of the workstations, companies had a conforming risk analysis while 37% of them had not carried out any risk analysis.

Among the 258 workstations, the return to work was possible for 41.5% (N = 107) of them. Among workers employed in companies that were assessed as having total knowledge of the OProMa, the return to work was possible for 72.2% of the workstations. This percentage decreases to 44.1% in companies that were assessed as having partial knowledge of the OProMa and to 32.7% in companies with little knowledge of the OProMa. Among the workstations of the companies that carried out a conforming risk analysis, the return to work was possible for 90% of them. Among the workstations of companies with a non-conforming risk analysis report, return to work was possible for 62.5% of them. Return to work was possible for 37.1% for workstations from companies that did not realize a risk analysis.

When return to work was possible, it took 27 days on average (18 days for the median). Conversely, for the workstations where return to work was not possible, the average time spent on preventive leave (i.e., duration until the delivery) was 133 days, with a maximum of 234 days (Table 3).

**Table 3:** Time in days spent in preventive leave until the delivery for those pregnant workers who did not return to work (N = 151).

Mean	Standard Deviation (SD)	Min	25th percentile	Median	75th percentile	Max
133.42	47.93	42	97	128	164	234

### Results of the regression models

Table 4 presents the results of the estimated regression models for the 2 variables of interest 1) the probability of returning to work and 2) time necessary to return to work.

**Table 4:** Results of regression models of the probability of returning to work and the number of days required to return to work.

Predictors	Probability of returning to work (Logistic regression)				Time to return to work (Zero-truncated negative binomial regression)			
	OR	SE	CI 95%	P value	IRR	SE	CI 95%	P value
(Intercept)	0.82	0.64	[0.17;3.83]	0.796	14.64	7.29	[5.39;44.21]	<0.001
**Women characteristics**								
Aged 19–25	1.33	0.52	[0.61;2.84]	0.466	0.53	0.12	[0.33;0.86]	0.006
Aged 35–46	0.97	0.37	[0.45;2.02]	0.926	0.99	0.24	[0.63;1.62]	0.976
Referred to the PregOH-consultation during the first 3 months of pregnancy	2.08	0.74	[1.04;4.20]	0.039	1.78	0.36	[1.20;2.68]	0.004
**Nature of risk exposure**								
Postures	1.08	0.74	[0.28;4.31]	0.907	1.51	0.63	[0.56;3.56]	0.328
Handling heavy loads	0.45	0.20	[0.18;1.10]	0.080	0.93	0.24	[0.55;1.52]	0.793
Chemicals	0.92	0.28	[0.50;1.68]	0.775	1.52	0.29	[1.04;2.19]	0.029
Microorganisms	1.27	0.38	[0.70;2.29]	0.425	0.69	0.13	[0.48;1.00]	0.050
Physical (cold, heat or humidity)	0.88	0.31	[0.44;1.74]	0.715	0.80	0.17	[0.53;1.21]	0.272
Noise	0.97	0.51	[0.34;2.70]	0.959	0.93	0.32	[0.49;1.90]	0.826
Vibration	0.38	0.22	[0.11;1.14]	0.100	1.00	0.42	[0.41;2.60]	1.000
Working pace	0.16	0.12	[0.03;0.64]	0.018	1.20	0.74	[0.34;5.15]	0.766
Ionizing radiations and Electromagnetic field	0.45	0.34	[0.09;1.86]	0.291	0.87	0.41	[0.37;2.39]	0.764
**Company characteristics**								
Total knowledge of the OProMa	3.56	2.24	[1.08;13.18]	0.043	1.00	0.30	[0.56;1.86]	0.990
Partial knowledge of the OProMa	1.56	0.46	[0.87;2.82]	0.136	1.14	0.22	[0.77;1.67]	0.504
Conforming risk analysis report	17.83	21.36	[2.56;405.26]	0.016	0.82	0.27	[0.42;1.66]	0.547
Non-conforming risk analysis report	2.75	1.43	[1.01;7.91]	0.052	1.66	0.46	[0.96;2.99]	0.069
Observations	258				107			
R^2^	0.152				0.296			
AIC	341.002				930.597			
log-Likelihood	-153.501				-447.299			

OR: odds ratio (exponentiated coefficient of the regression); IRR: incidence rate ratio (exponentiated coefficient of the regression); SE: standard error; CI: confidence interval. Robust standard errors are reported. Aged 26–34, none OProMa knowledge and not having performed any risk analysis are reference categories.

*Ceteris paribus*, the age of the pregnant workers is not significantly associated with the probability of return-to-work. However, the time needed to make adjustments is significantly associated with younger age. Workplaces held by younger women are significantly associated with a shorter time to accommodations (IRR for age = 0.53).

Women who were referred to the PregOH-consultation within the first 3 months of pregnancy were twice more likely to return to work than those who were refereed later (OR = 2.08). Among women who were able to return to work, early referral to the PregOH-consultation, is associated with a longer time to return to work compared to later referral (IRR = 1.78).

Regarding the type of occupational hazard faced by the pregnant workers, exposure to working pace is significantly associated with a lower probability of returning to work (OR = 0.16). The results for the other types of hazard exposure tend to show that exposure to a strenuous or dangerous activity is associated with a lower probability of returning to work, although the coefficients are not statistically significant. The type of hazard exposure is not significantly associated with time needed to make adjustments, except for chemicals and microorganism exposures. Workstations exposed to microorganism (or chemicals) are associated with a 31% reduction (resp. 52% increase) in time to return to work compared to stations not exposed to these hazards.

Workstations for which companies have a total knowledge of the OProMa are significantly associated with a three times (OR = 3.56) higher probability of returning to work compared to workstations for which companies have no knowledge of the OProMa.

Workstations for which employers have partial knowledge of the OProMa are not associated with a higher probability of returning to work compared to those for which companies have no knowledge of the OProMa. Companies’ knowledge of the OProMa does not affect the time needed to return to work.

After controlling for other variables, workstations for which companies have a conforming risk analysis report, rather than no risk analysis assessment, are associated with a higher probability (OR = 17.83) of returning to work.

Moreover, women working at workstations with a non-conforming risk analysis report have an increased probability (OR = 2.75) of returning to work compared to those working at workstations with no risk analysis. Regarding the time needed to return to work, the results also show that workstations with non-conforming risk analysis report are associated with an increased time (plus 66%) compared to workstations for which no risk analysis was performed.

## Discussion

Pregnant workers may interrupt their work for reasons related to illness, but also for reasons directly related to occupational hazards that endanger their health and that of the unborn child. This study addresses the issue of work interruptions due to occupational hazards among pregnant workers in a Swiss context. It focuses on the return to work of a group of pregnant women who stopped their activity because they were exposed to occupational hazards and no protective measures were taken by the employers. In accordance with the national Maternity Protection Ordinance, these pregnant women were given a certificate of preventive leave by their gynecologists to protect their health.

The first contribution of the study is to highlight the burden on companies and workers associated with pregnant workers’ absenteeism due to occupational hazards and lack of protective measures. In the study sample, among the 258 workplaces where pregnant workers were on preventive leave, we found a significant proportion of workers who did not return to work before the end of their pregnancy (58.5%). For these workplaces, the average period of incapacity to work was almost 4.5 months (133 days) during which the women were absent from work. This corresponds to 20,147 cumulative days of absence for the corresponding 144 pregnant workers. For the pregnant workers who returned to work (41.5%), the average time required to adapt the workplace and return to work in safe conditions varied from 1 day to 168 days, with a median of 18 days. The 107 workstations for which a return to work was possible represent a total of 2,859 cumulative days of absence for pregnant workers. Overall, these absences are substantial for both employers and employees. In Switzerland, there is no prenatal leaves [32] and women are expected to work up to the time of childbirth if they wish to continue to receiving their full salary. Absence from work during preventive leave is financially supported by the employer, who must pay the employee compensation equal to 80% of her salary. In other countries, the employer must also pay all or part of the income during preventive leave. For example, in the United Kingdom, the employer must pay 100% of the salary during preventive leave [33] and in Quebec, the first 5 days of preventive leave are fully paid by the employer [34]. In general, this situation is different from sick leave, which requires a certificate of illness and is financed by dedicated insurance in most of the countries. In addition to the direct loss to employers, absence from work requires organizational and human resource management to replace and schedule work. The financial burden associated with pregnant employees on preventative leave may have a greater impact on companies compared to those on sick leave. Pregnant workers may also be financially affected by the absence from work associated with a medical certificate of incapacity, as they face a loss of wages. In the specific context of Switzerland, this loss can reach 20% of the salary [35]. In Quebec, it is 10% [34]. Additionally, the potential consequences are not limited to monetary loss. Forced to stay at home, pregnant workers may feel excluded from the workplace and feel guilty for not fulfilling their obligations to employers and even colleagues [36]. In addition, work opportunities may be lost and women’s careers may be affected [37].

We did not find any evaluation of the costs associated with the absences of pregnant workers in the literature. Yet, measuring the costs of absenteeism caused by health conditions is of great interest and a challenge in many countries. The analyzed absenteeism, which generates direct, indirect and hidden costs, is generally caused by health problems and results in a considerable burden for several stakeholders, including employers, employees, insurance companies and society as a whole. At European level, the annual national cost of absences is estimated to be around 2.5% of gross domestic product in 2010 [38]. Reducing absenteeism benefits employers, workers, insurers and society as a whole. In the specific situation of preventive leave for pregnant workers, this is even more true as the absences are not related to the worker’s illness, but to the employer’s compliance and application, as far as possible, of maternity protection legislation in the workplace.

The second contribution of this study is to provide information on some of the predictors of return to work in safe conditions for pregnant women that have been prescribed preventive leave. Taking into account employee and employer characteristics, the multivariate regression models allow the identification of factors associated with return to work. Compared to women who are referred later, the earlier in the pregnancy the workers are referred by their gynecologists to the PregOH-consultation, the higher the probability of returning to work in safe conditions. From the employer’s point of view, the longer a pregnant woman has to work before giving birth, the greater the incentive to make workplace adjustments. First, absenteeism represents an organizational burden and loss of productivity for the company. Second, the employer is financially responsible for 1) the cost of the job adjustment and 2) the payment of employee’s salary while the pregnant employee is on preventive leave. It is in the employer’s interest to reduce the period during which this amount is paid to an employee who is not working. In addition, the shorter the period of preventive leave, the more cost-effective the investment in adaptation measures will be. Conversely, the later in the pregnancy the visit to the PregOH-consultation takes place, the less incentive there is for the employer to make adaptations. Delayed action by the pregnant woman herself and her gynecologist also reduces the window of opportunity to implement workplace protective measures during pregnancy. These findings underscore the importance of having proactive behavior on the part of pregnant women and gynecologists in accessing information and becoming aware of their rights as workers in the workplace. Even if it is not mandatory for pregnant workers to inform their employer of their pregnancy, an early dialogue with the employer seems to be essential in order to take protective measures. Moreover, in the case of return to work, a late referral to the PregOH-consultation is associated with a shorter time used for adaptations. In this later case, work adaptations could focus more on offering safe alternative workstations during pregnancy than on implementing different protective measures when there is little time left before childbirth. This choice of protection against risks can be implemented more quickly, especially for the biggest companies. This is an alternative that should not be overlooked by either the employee or the employer.

The age of the pregnant women appears to affect their return to work. The job type and the position in the company of younger women may be easier to adapt, making their return to work faster than older women. As we controlled for risk exposure, this faster return to work could also be due to different employer attitudes. Employers may have more incentives to make adjustments for younger pregnant workers than for older ones. Changes caused by pregnancy or medical complications, which are sometimes age-related and lead to potential work interruptions, may be less common among younger women [39, 40]. Thus, once accommodations are made, employers may expect younger women to work longer. It would then be more cost-effective from an employer’s perspective to adapt the workplaces occupied by younger pregnant workers.

The results show that an intensive pace at work tends to reduce the probability of returning to work by 84%. This result seems to be consistent with findings on the impact of high work intensity on health and well-being analyzed among non-pregnant workers. Indeed, in the literature, a high pace at work is associated with high levels of stress and fatigue, and lower job satisfaction [41]. In addition, work intensity is a strong predictor of adverse health outcomes, and blue-collar workers would experience higher levels of work intensity compared to other types of occupations [41]. Moreover, in companies where work intensity is important, there may be little scope for either reducing the working pace or offering alternative work to the pregnant workers. This is likely to be the case, for fitters, packers and assemblers working in manufacturing industry, and who account for about 60% of the pregnant women with such exposure in our sample.

The workstations with chemical exposures require more time to implement adaptations, if possible. This may be explained by the specific nature of this exposure, which requires a thorough analysis of the specific chemical substance and for which the implementation of adaptation measures is complex task. Conversely, *ceteris paribus*, time to make work adjustments in the context of exposure to microorganism is shorter. Microorganisms are bacteria, viruses, fungi or parasites that can cause infectious disease that are dangerous for the pregnant worker and her future child. This was particularly the case for exposure to cytomegalovirus and for childcare and healthcare workplaces [42], which accounted for a significant proportion (64%) of the microbial exposure situations in our sample. The adaptions in these workplaces consist of targeted standard hygiene rules, the wearing of individual protection (mask and gloves) and the substitution of work assignments in the older children’s sector rather than in the younger ones. This kind of workplace adaptations have been shown to limit the risk of disease [43, 44] and can generally be made in relatively short period of time. Regarding our sample, exposures to microorganisms also concerned COVID-19 exposures in different sectors of activity. During the epidemic, the regulations and guidelines for pregnant women in Switzerland evolved considerably over time. While the first months of the pandemic, pregnant women were not included in the category of vulnerable persons requiring enhanced protective measures, their vulnerability was recognized in August 2020 [45]. The guidelines encouraged then gynecologists to use preventive leave to protect pregnant workers exposed to the virus. It is likely that protective measures didn’t require much time since that date because employers were better informed.

Interestingly, after controlling for the characteristics of the pregnant workers and the working conditions, our results indicate that the characteristics of the employer play a significant role, with the presence of a risk analysis report (conforming or non-conforming) and the employer’s total knowledge of the OProMa found as strong predictors of the pregnant workers’ return to work in safe conditions. Identifying the hazards and the necessary adjustments to be made before the pregnancy is announced—through a risk analysis—allows the company to be more proactive in terms of job adaptations and thus increasing the likelihood of a return to safe work.

Among pregnant workers who have safely returned to work, the extra time associated with a non-conforming risk analysis can be attributed to the following reasons: 1) The PregOH consultation’s occupational physician needs time to identify and correct the deficiencies in the document that do not meet the standards. And 2) it’s challenging for the occupational physician to persuade the company to make the necessary adjustments. It can be more complex if the help and assistance come from outside the company. The occupational physician may not visit the company in person to resolve the issue but make suggestions through documents. Additionally, other unidentified factors like the employer’s stance towards the law or the worker may also impact the situation.

As far as we could find, the only study that examined the issue of return to work per se among pregnant employees is that of Kristensen et al. [23] in a Norwegian context. While the authors focused on the relationship between job adjustments and absences from work, they also examined the probability of returning to work in week 30 among pregnant workers who were absent in week 17. The main determinants considered were a classification of adjustments of working conditions (not needed, needed and obtained, and needed but not obtained), job characteristics, and work environment. The results of the analysis, conducted on a sample of 5679 employed women indicated that, compared with women who needed job adaptations but did not receive them, obtaining job adjustments was associated with a 4.1% increased probability of returning to-work. After controlling for job adjustments categories, the probability of returning to work was associated with favorable work environments. The effects of individual characteristics such as age, origin and educational level on the probability of return to work were not investigated. Our approach differs from that of Kristensen et al. [23] in terms of the explanatory variables examined as affecting the probability of returning to work. In addition, the Norwegian study did not examine the time needed to make adjustments. The occupational risk variables in the Norwegian study were collected through a questionnaire completed by the pregnant workers, whereas in our study they were objectively assessed by professionals. It is therefore difficult to compare the results of both studies. However, good working conditions consistently predict the likelihood of pregnant workers returning to work in both studies. In addition, our study highlights that company characteristics can also play a significant role in the return-to-work process. Companies’ preventive measures and increased knowledge of the law favor the chances of returning to work in safe conditions.

The implementation of workplace accommodations during pregnancy can be challenging for the various stakeholders involved, including pregnant women, and companies [5]. Pregnant women and employers may not agree on what needs to be done to keep pregnant women safe, due to different views on occupational risks [46]. If the company thinks the job is safe, they might not take steps to protect workers [47]. Therefore, the employer should mandate an occupational physician or other occupational health specialist to visit the company and conduct a risk analysis. By doing so, everyone involved would have a shared vision of the dangerous and arduous activities and the protective measures that should be implemented.

At the company level, making changes to support pregnant employees can be a slow process because it requires the whole organization to change the way it handles pregnancy and occupational health. This is especially true if the company has not established a culture of health and safety for all workers. Depending on how much the companies anticipate the implementation of measures to protect pregnant workers, work accommodations could either help or limit the activity. At the institutional level, there may be many practical barriers to imposing the implementation of accommodations on organizations [5]. We did not find any cost-benefit analysis of the implementation of workplace accommodations by companies during pregnancy to reduce absenteeism. However, human and financial investments in workplace accommodations could be beneficial for both employees and employers in terms of improving working conditions and reducing absenteeism. By providing monetary values, the results of such analyses could also help the implementation of workplace accommodations aimed at reducing absenteeism.

The interest in such analyses is reinforced by the results of studies suggesting that there are longer-term effects of adverse working conditions on absenteeism among women of childbearing age, i.e. beyond the period of pregnancy. Few studies emphasize that the work environment experienced during pregnancy plays a role in women’s return to work after childbirth, i.e., after the maternity leave period [48]. In an attempt to identify factors associated with return to work within the first year postpartum among women who worked during pregnancy, Wallace et al. [48] showed that, after controlling for socioeconomic and health variables, occupational factors were significantly associated with return to work. In particular, women whose jobs did not require constant standing were more likely to return to work. A recent analysis conducted in Thailand also examined factors associated with postpartum return to work for a cohort of mothers who were actively employed at the beginning of their pregnancy [49]. About 90% of the 9,369 women surveyed returned to work before their full-paid maternity leave ended. Women experiencing higher job stress during pregnancy were 1.72 times more likely to return to work later, specifically between 6 and 12 months compared to those with a less demanding workload.

### Limitation and strength

Our analysis has some shortcomings. The data analyzed in the present study are information related to work situations that were referred to the PregOH-consultation. Since the referral to the consultation is made by the gynecologists, it is likely that these work situations are complex situations that could not have been resolved by the companies themselves and for which the support of an external and additional occupational physician, was needed. As a result, the work cases examined in the context of this study do not reflect the average of work situations. This limits the transferability of the results. Second, the sample size is small compared to the total number of births (60’483 live births) during the study period (2015–2021) in the canton of Vaud [50]. The study sample is therefore not representative of pregnant workers in French-speaking Switzerland. Further analyses on a larger scale and other settings are required. However, the strength of this study lies in its contribution to the still scarce literature, on maternity in the workplace, and the relationships between work absences, workplace accommodation, return to work and their determinants. To the best of our knowledge, this is the first study using a quantitative approach/multivariable model that addresses this issue by examining both the determinants of the probability of returning to work and the determinants of the time required to make the adjustments that allow a return to work in safe conditions.

## Conclusion

Maternity in the workplace is a major public and social concern in OECD countries. Legislation on maternity protection in the workplace provides for the implementation of special measures to enable women to continue working in safe conditions during pregnancy. In practice, these measures may not be fully applied. For some pregnant women, this means stopping work and taking preventive leave. More research is needed on this issue. This study highlights the burden on companies of absenteeism among pregnant workers exposed to occupational hazards and on preventive leave. It also sheds light on the patterns and predictors of return to work among these pregnant workers. The results underline the interest, for companies, to invest in the prevention of occupational risks and their application in order to reduce absenteeism and its duration. Companies with complex working conditions may have an even greater interest in anticipatory measures to limit the costs of absenteeism among pregnant workers. This study also provides potential avenues for interventions aimed at improving the maternity protection process.

## Supporting information

S1 Appendix OproMa knowledge criteria. (DOCX)
