# Supplementary material for: Predictors of the return to work for pregnant employees on preventive leave: Patients from an occupational medicine consultation in Switzerland
Source: PLoS One. 2024 Mar 22;19(3):e0300686. doi: 10.1371/journal.pone.0300686 (PMC10959330; doi:10.1371/journal.pone.0300686)
Supplement: S1 Appendix — (DOCX) [file pone.0300686.s001.docx]

**Supportive information**

**S1: OProMa knowledge criteria**

During a phone call, the occupational health physician of the PregOH-consultaion submitted questions to the employers in order to evaluate the degree of the OProMa knowledge regarding the following 7 criteria:

Major criteria:

- The employer knows the OProMa law.
- The employer knows what a risk analysis is.
- The employer knows that the gynaecologist can deliver a work incapacity medical certificate.
- The employer knows what the consequences of the work incapacity medical certificate are (the pregnant worker must not work, goes on preventive leave, and he has to pay 80% of her wage during the absence)

Minor criteria:

- The employer knows that he must adapt the workplace of the pregnant women to preserve her health.
- The employer knows that he can offer an alternative workplace to preserve the health of the pregnant woman.
- The employer knows what the activities at risk according to the OProMa are.

To create the variable, the coder uses the following rules:

- “No knowledge”: No criterion (0 major, 0 minor). It means that the employer does not know that the pregnant woman has specific rights, and does not know the existence of OProMa.
- “Partial”: All possible combinations with at least one major or one minor criteria, excluding 4 majors. It means that the employer knows a part of the rights of the pregnant worker but does not know all the 4 majors criteria.
- “Total”: 4 criteria of major knowledge (regardless the number of minor criteria).
